# The Ozkan Technique in Current Use in Uterus Transplantation: From the First Ever Successful Attempt to Clinical Reality

**DOI:** 10.3390/jcm12082812

**Published:** 2023-04-11

**Authors:** Omer Ozkan, Ozlenen Ozkan, Nasuh Utku Dogan

**Affiliations:** 1 Department of Plastic Surgery, Faculty of Medicine, Akdeniz University, 07070 Antalya, Turkey; 2 Department of Gynecology, Faculty of Medicine, Akdeniz University, 07070 Antalya, Turkey

**Keywords:** uterus transplantation, deceased donor, repeated pregnancy losses, venous supercharging, live birth

## Abstract

Uterus-related infertility affects 3–5% of all young women, including Mayer-Rokitansky-Küster-Hauser (MRKH) syndrome, hysterectomy, or severe Asherman syndrome. For these women with uterus-related infertility, uterus transplantation is now a viable option. We performed the first surgically successful uterus transplant in September 2011. The Donor was a 22-year-old nulliparous woman. After five failed pregnancy attempts (pregnancy losses), ET attempts were discontinued in the first case, and a search for underlying etiology was performed, including static and dynamic imaging studies. Perfusion computed tomography revealed an obstructed blood outflow, particularly in the left anterolateral part of the uterus. In order to correct blood flow obstruction, a revision surgery was planned. By laparotomy, a saphenous vein graft was anastomosed between the left utero-ovarian and left ovarian vein. Perfusion computed tomography performed after the revision surgery confirmed the resolution of venous congestion and a decrease in uterine volume as well. Following surgical intervention, the patient was able to conceive after the first embryo transfer attempt. The baby was delivered with cesarean section at 28 weeks’ gestation due to intrauterine growth restriction and abnormal Doppler ultrasonography findings. Following this case, our team performed the second uterus transplantation in July 2021. The recipient was a 32-year-old female with MRKH syndrome, and the donor was a 37-year-old multiparous braindead woman due to intracranial bleeding. After the transplant surgery, the second patient experienced menstrual bleeding six weeks after the operation. Seven months after the transplant, in the first ET attempt, pregnancy was achieved, and she delivered a healthy baby at 29 weeks of pregnancy. Uterus transplantation from a deceased donor is a feasible option for treating uterus-related infertility. When confronted with recurrent pregnancy losses, vascular revision surgery via arterial or venous supercharging could be an option in order to deal with focal underperfused areas defined by imaging studies.

## 1. Introduction

Uterus-related infertility affects 3–5% of all young women, including Mayer-Rokitansky-Küster-Hauser (MRKH) syndrome, hysterectomy, or severe Asherman syndrome [1]. Surrogacy and adoption are alternative methods, but until now, there was no treatment for those who desired to have their own (genetically related) children. Surrogacy is prohibited in many countries, and adoption does not establish a genetic link between mother and child. The first attempt from Saudi Arabia failed on the 99th day, probably from vascular obstruction of the graft [2]. The first surgically successful uterus transplantation (UTx) was performed in Turkey in September 2011 [3]. Following this first surgically successful attempt and livebirth after living donor UTx in Sweden in 2014 [4], more than 80 UTx have been performed from either living or deceased donors, and more than 30 live births have been reported [3,4,5]. In this review, we described the surgical procedures, previous failed pregnancies, methods for overcoming pregnancy failure, and most importantly, the birth of a healthy infant in the first successful UTx from a deceased donor. We also summarized briefly the second case performed in our unit.

## 2. The First Uterus Transplant: Case 1

The case performed by our team in Turkey was the first successful transplant in the world and only the second procedure after the failed attempt in Saudi Arabia [2,3]. The first recipient was a 21-year-old female with MRKH syndrome. She was operated on for vaginal reconstruction using the jejunal segment two years before the surgery. The kidney function tests and renal anatomy were normal. She was a healthy woman with a BMI of 21 kg/m^2^. The donor was a 22-year-old nulliparous braindead woman. Blood group type; human leukocyte antigen (HLA) matching; and screening for toxoplasma, rubella, cytomegalovirus (CMV), human immunodeficiency virus (HIV), and hepatitis B and C were all pretested. The transabdominal ultrasound was normal, without any structural abnormalities. Before the surgery, in the initial preparatory phase, approval from the local transplantation committee and the institutional review board were obtained for all potential candidates with MRKH syndrome. The inclusion criteria for the candidates were as follows: normal 46 XX karyotype; age 21–40 years; sufficient ovarian reserve (detected by follicle-stimulating hormone (FSH), estradiol (E2), antimullerian hormone (AMH) levels, and antral follicle count); vaginal length >5 cm; body mass index <25 kg/m^2^; previously retrieved 5 < good quality embryos; presence of 3 years of stable marriage status; and good social support. The exclusion criteria were also as follows: presence of hypertension; diabetes mellitus; acquired or congenital thrombophilia; cardiac, renal, or central nervous system diseases; psychiatric disorders; seropositivity for HCV, HBV, or HIV; alcohol and drug abuse; cigarette smoking; renal abnormality, including pelvic or low-lying ectopic kidneys; previous multiple abdominal surgeries; history of severe endometriosis; and history of any genital malignancy. 

## 3. Surgical Technique

**Figure 1:**
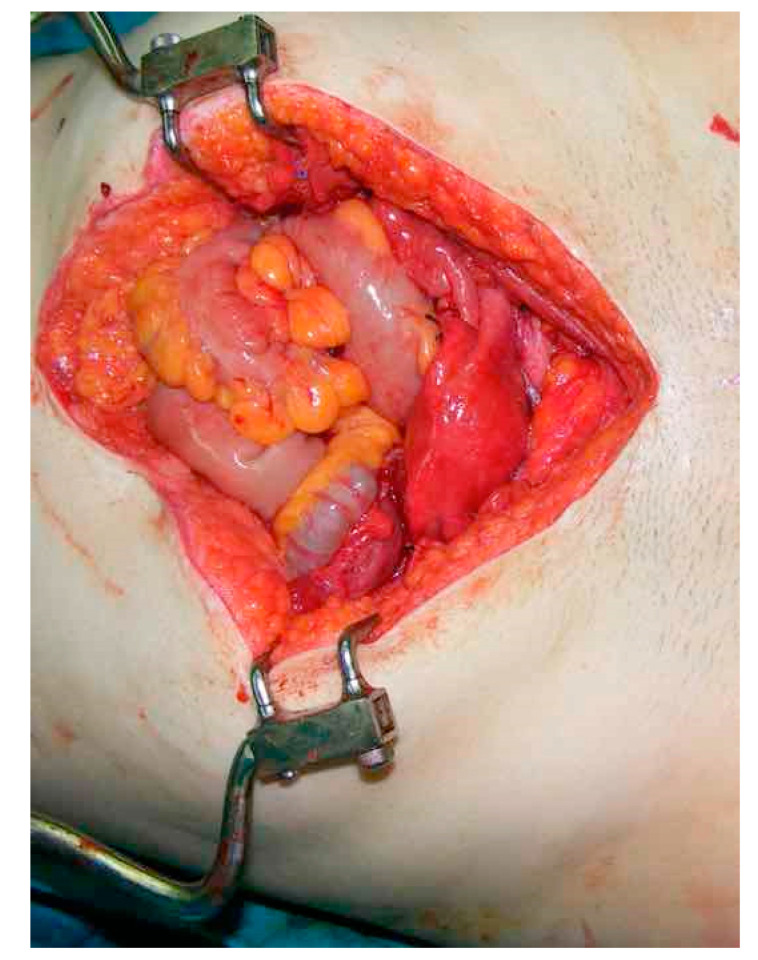
Situs after transplantation of the uterine graft.

We retrieved the uterine graft as the first procedure before the retrieval of other organs and by this way we were able to have relatively long vascular pedicles of common iliac artery and veins from the donor. The retrieval process and transfer time were 120 and 30 min, respectively. Regarding the recipient surgery, a midline incision was performed. The external iliac artery and vein were dissected. Following back table preparation of the graft, the donor uterus was transplanted in the orthotopic position. The donor vagina was sutured to the proximal side of the jejunal recipient neovagina. Sacrouterine ligaments were sutured to the sacrum and by this way a three-dimensional neo-uterus was achieved. Following this step, anastomosis of vascular pedicles was performed as end-to site anastomosis between the recipient’s external iliac vessels and the donor’s hypogastric vessel pedicle. Regarding back table preparation and uterine graft positioning, the length of donor uterine vessel pedicle was adjusted as a smooth curve and by this way a tension free anastomosis was created. Following vascular anastomosis, round ligaments were sutured to inguinal ligament of the recipient. Anterior bladder peritoneal flap retrieved along with donor uterus was sutured to the parietal peritoneum of the recipient. Moreover, bilateral salpingectomy was performed. The fixation of the anterior bladder flap is a crucial part of the procedure to hold the uterine graft in an anatomical position (Figure 1). We described recently this part of the surgery and published details of the procedure (Ozkan uterus transplant technique). This technique was also adopted by the Swedish team who visited our clinic after our successful UTx procedure [1,3]. Following the declamping of vessels, the perfusion of the graft was observed with a color change from pale yellow to red. The blood flow was checked by an intraoperative color Doppler ultrasonography. Moreover a rectangular sentinel skin graft from the donor was retrieved and positioned onto the anterior thigh of the recipient to observe and monitor any possible rejection.

## 4. Immunosuppression Regimes

Intraoperatively, the induction was initiated with thymoglobulin (2.0 mg/dL). Thereafter, the dose was given based on the CD3 levels for 10 days. Moreover, 1000 mg prednisolone was given intraoperatively and then continued with 20 mg for one week following the surgery. Tacrolimus was administered with a dose of 0.2 mg/kg starting on the seventh day postoperatively. Fluconazole, piperacillin/tazobactam, cotrimoxazole, nystatin drops, and valacyclovir tablets were given for the purpose of prophylaxis for ten days after the surgery. As maintenance, tacrolimus, mycophenolate mofetil, and prednisolone (20 mg/day) were given. Low molecular weight heparin was given for thromboprophylaxis.

## 5. Follow-Up and Monitorization of the Transplanted Uterus

Doppler ultrasonography of uterine arteries were performed to confirm the arterial patency beginning from the immediate postoperative period. In the first three months, vaginal (donor) biopsy was used to monitor for acute rejection every two weeks. The first menstrual cycle was observed three weeks after the surgery, and the first three cycles was not regular. Chemical pregnancy was observed after the first embryo transfer one and a half years after the transplant. An increase in hCG levels (up to 35 IU/L) was detected, but there were no ultrasonographic sign of pregnancy. After this chemical pregnancy, the patient had an abortus (7 weeks) in June 2013. Then, the patient had 3 unsuccessful embryo transfer (ET) attempts in 2014 followed by another abortus (7 weeks) and 3 more failed ET attempts in 2015. The patient experienced abortus at 8 weeks in the tenth ET attempt in 2016 and another pregnancy loss at 8 weeks in twelfth ET in 2017. Following five pregnancy losses, the ET attempts were discontinued and a search for underlying etiology was performed. Static magnetic resonance imaging of the uterus revealed anatomically normal uterus with a normal blood flow. In order to see the dynamic arterial and venous blood flow of the transplanted uterus, a perfusion computed tomography was planned. An obstructed blood-outflow, especially in the left anterolateral part of the uterus was observed in perfusion computed tomography (256-slice helical SOMATOM Definition Flash CT, Siemens Medical Solutions, Erlangen, Germany). We previously published the details of radiologic work-up of the present case [6]. To overcome the obstructed blood-outflow, an exit plan to restore venous outflow from the uterus to the central venous pool was decided. Vascular venous revision was planned and official permission from Turkish Ministry of health and approval from Akdeniz University Ethical committee were obtained. We used a saphenous vein graft between one of the branches of the left utero-ovarian veins (end-to-end) and the left ovarian vein (end-to-side). Following vessel anastomosis, the left ovarian was clamped and end to side anastomosis was done. The total clamping and anastomosis time was 15 min. After the anastomosis, the arterial and venous patency was evaluated by Doppler ultrasonography. The anastomosis worked without any problems following the surgery confirmed by Doppler ultrasonography. Perfusion computed tomography performed after the revision surgery showed the resolution of venous congestion on the fundal side and a significant decrease in uterine volume (compared to the preoperative period). Three months after the surgery, three cycles of controlled ovarian stimulation and oocyte pick-up was performed. Oocytes were fertilized by ICSI. PGT-A was performed by NGS technology (Igenomix; Valencia, Spain). 

**Figure 2:**
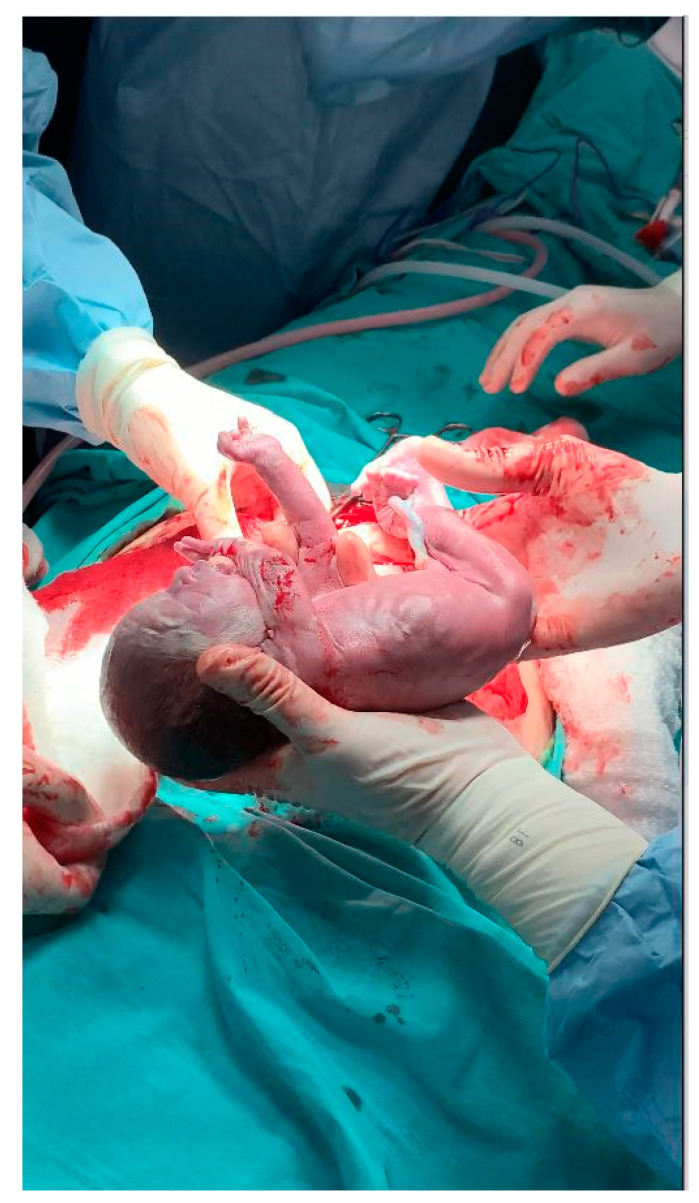
Birth of the baby from first successful uterus transplant.

**Figure 3:**
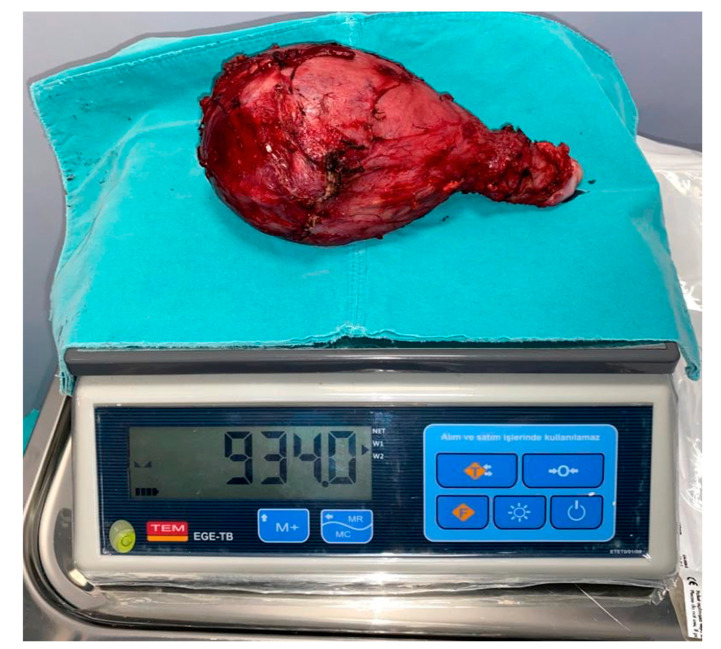
The uterine graft following cesarean hysterectomy.

On the first embryo attempt following the revision surgery, the patient was able to conceive, and the serum β-hCG level was measured to be 923 IU/L two weeks after the 13 days after the 14th embryo transfer. Fetal cardiac activity was checked and seen 28 days after the embryo transfer. The immunosuppressive treatment was modified to prednisolone, azathioprine, and tacrolimus and maintained with these regimes all through the pregnancy. In the course of pregnancy, we planned to take cervical biopsies in each trimester, but because of the risk of infection related to preterm rupture of membranes, we did not perform any cervical biopsy. We rather performed serial speculum examination of the cervix and also took serial complete blood counts and looked for clinical signs of rejection. Moreover, routine follow-up of sentinel skin graft from the donor implanted on the recipient’s anterior thigh let us observe possible rejection episode. To note, the patient experienced no clinical rejection episode in the period from UTx to hysterectomy. In the late second trimester, we observed beginning of intrauterine growth restriction and abnormal findings in Doppler ultrasonography. With these findings, we planned cesarean section on the 28 weeks of gestation. Due to the long immunosuppressive period, hysterectomy was discussed as an option during cesarean section, and she opted to have her uterus removed during surgery. On 4 June 2020, a 760 g male fetus with Apgar score of 7-8-8 was delivered (Figure 2 and Figure 3). After the surgery immunosuppression treatment was tapered and finally stopped on the postoperative seventh day. After delivery, the baby was admitted to NICU. On the 79th day of admission, the baby was successfully discharged from the NICU, weighing 2475 g. It has been two and a half since the birth of baby. Both mother and baby were healthy without any morbidity. The renal functions of the mother were normal during the pregnancy period and in the last follow-up as well. 

## 6. Case 2

After the birth of the baby from the first surgically successful UTx recipient, the second UTx was performed in our unit in July 2021. Before the transplantation procedure, in the preparatory period, the patient was evaluated by our reproductive endocrinology specialists for parameters that may affect the embryo quality. Female AMH level was 13 ng/mL. Serial hormonal analysis results revealed that the patient was anovulatory. The male and female karyotype analysis were normal; 46 XY and 46 XX, respectively. Male factor was evaluated by standard semen analysis, which revealed normal sperm concentration, motility, and morphology. Oral contraceptive pills were started on a random day and used for 21 days. Gonadotrophin treatment (225 IU, Gonal-F (Merck)) on the fifth day of stimulation was started. During the stimulation, GnRH antagonist was administered with a fixed dose of gonadotrophins. On the tenth day of stimulation, the GnRH agonist trigger was performed (0.2 mg triptorelin (Gonapeptyl-Ferring). Thirty five hours later transvaginal ultrasound guided oocyte pick-up procedure was performed, and 16 oocytes were retrieved: 15 were mature and 12 were fertilized. Four blastocysts were biopsied for PGT-A and 2 were euploid. The donor was a 37-year-old multiparous braindead woman due to the intracranial bleeding. She was pretested for blood group type, human leukocyte antigen (HLA) matching, and screening for toxoplasma, rubella, cytomegalovirus (CMV), human immunodeficiency virus (HIV), and hepatitis B and C. The recipient was a 32-year-old female with MRKH syndrome. She had a vaginal length of 6 cm established previously by frank dilatation. 

**Figure 4:**
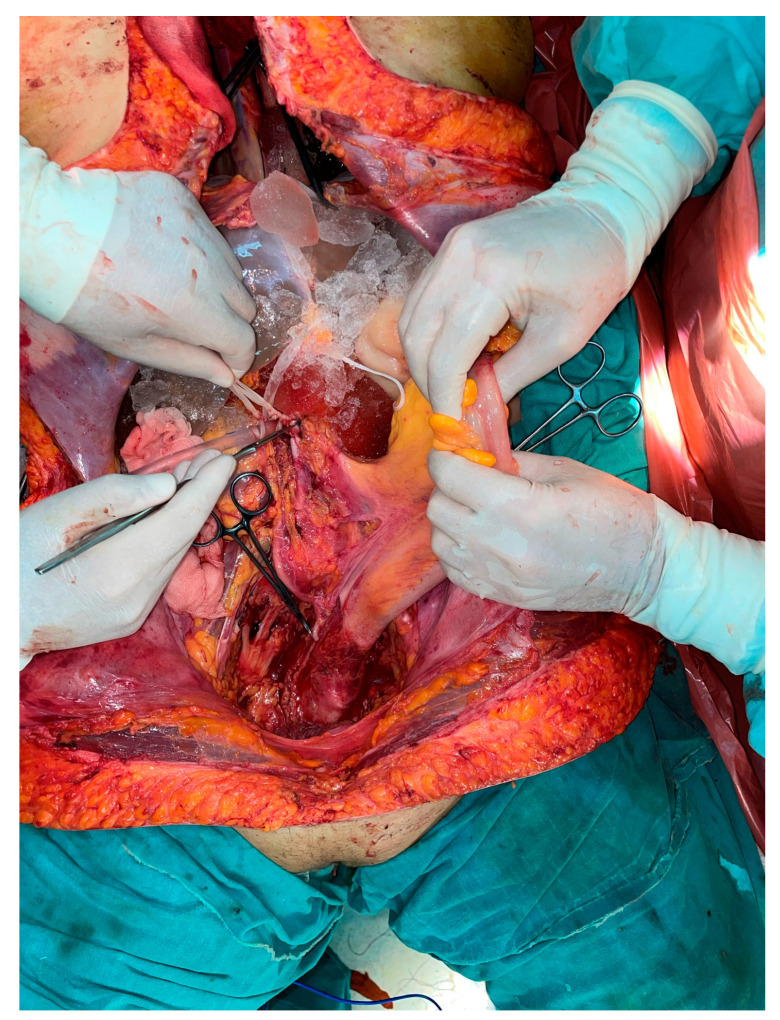
Situs after removal of uterine graft from donor. The deep pelvic structures are visible.

**Figure 5:**
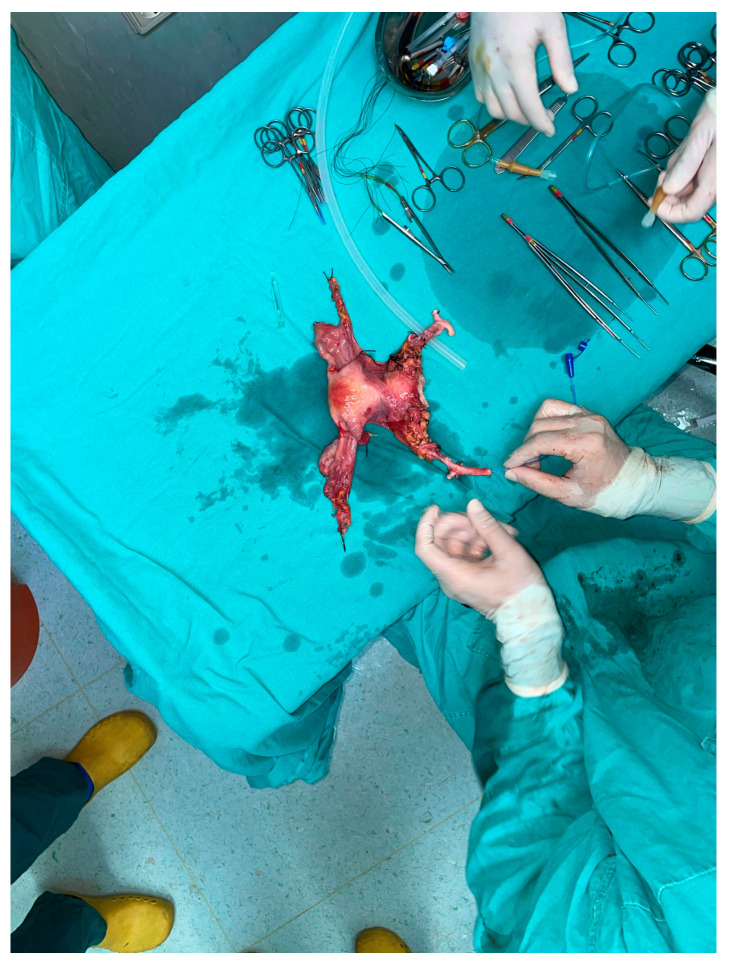
Uterine graft during back table preparation. Note the long vascular pedicles.

**Figure 6:**
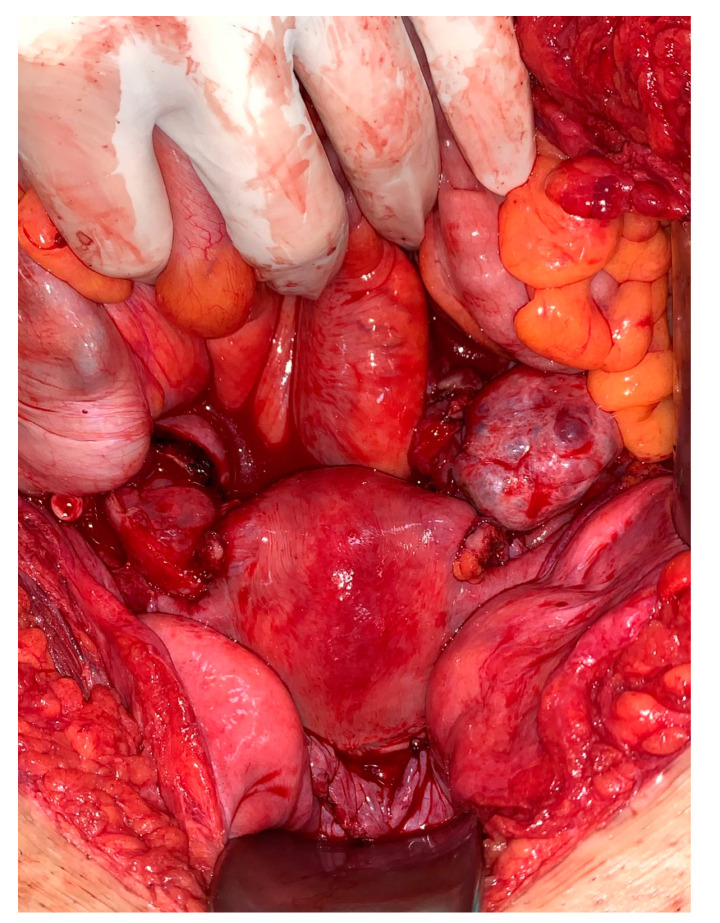
Uterine graft after the anastomosis of both uterine arteries and veins and ovarian veins. The red color of the graft was visible after removal of the bulldog clamps.

For the surgical technique for cadaveric retrieval procedure, uterus dissection was started as the first step. Then lungs, heart, and liver retrieval were carried out. After the retrieval of mentioned organs, hysterectomy for the uterine graft was performed as the last procedure. Long vascular pedicles of common iliac artery and veins were retrieved. This surgical procedure was different from our first case. In our first case, hysterectomy was done as the first surgical step in the cadaveric donor surgery before the retrieval of other organs. For the donor surgery, we performed similar surgical technique as the first case except anastomosis of two ovarian veins (right and left) to both external iliac veins (Figure 4, Figure 5 and Figure 6). The total ischemia time was 90 min and re-warming ischemic time was 75 min. Anastomosis duration was 30 min. In the postoperative twelfth hour, the patient was reoperated on due to the suspicion of intraabdominal bleeding. Hematoma in the pelvis was revealed and evacuated. The postoperative period was uneventful, and she experienced regular menstrual bleeding beginning from six weeks after the operation. We followed the same immunosuppression protocol as the first case including thymoglobulin for ten days. As maintenance, tacrolimus, mycophenolate mofetil, and prednisolone were given. A vaginal biopsy was taken to reveal acute rejection every two weeks in the first postoperative three months. We experienced one episode of mild rejection in the third month after the transplantation surgery. A pulse steroid was given, and resolution of the rejection was observed by vaginal biopsy. 

**Figure 7:**
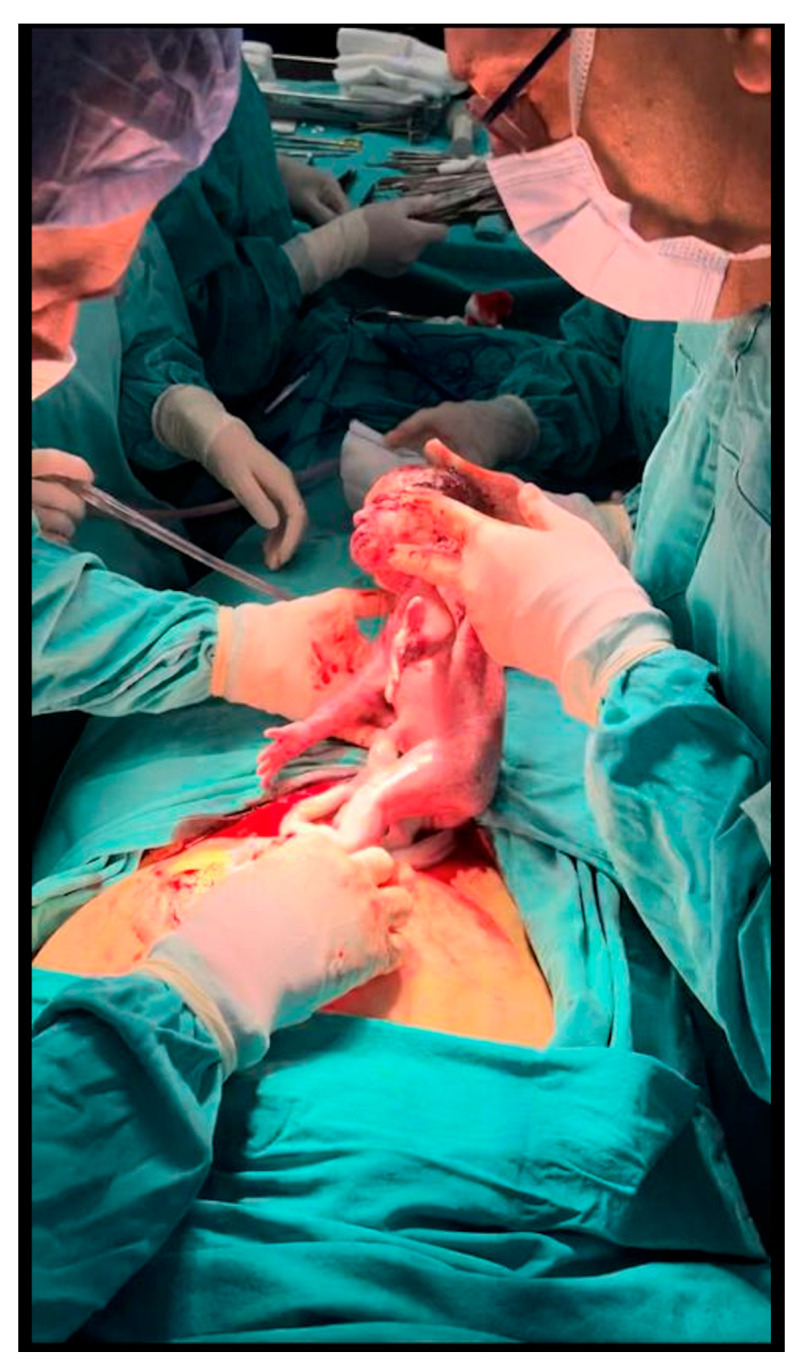
Birth of the second baby from the second uterus transplantation in Turkey.

After the transplantation, the patient had spontaneous regular menstruation. Six months after the transplantation procedure, endometrial preparation for embryo transfer (ET) was initiated and involved hormone replacement treatment. Oral estrogen (E) (Estrofem, Novo Nordisk, Istanbul, Turkey) administration was employed according to a step-up regimen: 4 mg/day on days 1–4, 6 mg/day on days 5–8, and 8 mg/day on days 9–12. Endometrial thickness was measured by transvaginal ultrasound (TV-USG) on day 12; and the endometrial thickness was 9 mm, E2 was 322 ng/mL, progesterone (P) was 0.12 ng/mL, and P was initiated as a once daily 100-mg IM-P injection (Progestan, Kocak Farma, Istanbul, Turkey). Single euploid ET was performed 120 h later with transabdominal USG guidance. The serum β-hCG level was measured 12 days after ET and revealed to be positive. Oral E replacement was stopped on week 6 of gestation, whereas P was continued until 10 weeks. The immunosuppressive treatment was changed to prednisolone, azathioprine, and tacrolimus all through the pregnancy. In the 28 weeks of gestation, she experienced a preterm premature rupture of membranes (PPROM). Antibiotics were initiated and corticosteroid was given to accelerate fetal pulmonary maturation and to avoid pulmonary morbidity. Following PPROM, she experienced regular contractions and had vaginal bleeding. Due to PPROM, vaginal bleeding and regular contractions, we proceeded with cesarean section on 29 weeks of gestation. The patient delivered 1720-g female healthy baby with Apgar score of 7–8–8 at 1–5–10 min respectively (Figure 7). As the general health of the baby was good, we performed cesarean hysterectomy. The baby was admitted to NICU. At postnatal 47 days, the baby was discharged from NICU, 2910 gr in weight. 

## 7. Discussion

In this manuscript, we described two uterus transplantations performed in our unit from deceased donors. The first case described here possessed unique characteristics such as inclusion of deceased donor and live birth after failed attempts successfully managed with the use of special exit strategy that was never performed before, namely, the use of vein graft in order to compensate venous outflow [3,6]. After our leading pioneer case, the other groups performed UTx and achieved live births. More than 80 UTx were reported, and more than 30 infants were delivered [7]. By the recruitment of uterus from deceased donor, the risk to donor decreases to zero and retrieval of long vascular pedicles is possible, thus the chances of successful vascular anastomosis increase significantly. The initial surgeries for organ retrieval from live donors in Sweden took more than 10 h, and ICU admissions for the donors were required [8]. Recently, donor surgeries using minimal invasive techniques such as laparoscopic and robotic platforms were all used in uterine retrieval surgery, but still with significant long operative durations [9]. Moreover, the donors experienced serious complications such as ureteric injuries, ureterovesical fistula formation, and vaginal cuff dehiscence [8,10]. In some series, in order to take longer vascular pedicles, oophorectomies were performed in premenopausal donors. Surgical menopause in these women has a possible risk of increased cardiovascular morbidity and increased risk of mortality [11,12]. With the above-mentioned drawbacks, UTx by deceased donor transplantation may have clear advantages such as avoidance of potential harm to the donor and increased chance of successful vascular anastomosis by taking longer vascular grafts. 

In our first case, the patient experienced five failed pregnancies. The basic work up for recurrent pregnancy losses revealed no pathology but in perfusion computed tomography study, a possible congestive area consistent with venous pooling was revealed as previously reported [6]. The resolution of this congested area, a decrease in the size of the uterus compared to preoperative period and successful pregnancy and birth after the revision surgery could be all be solid evidence of a real venous insufficiency. In UTx surgery, as far as we know, this is the first case in which additional revision surgery resulted with a live birth. In previous series, a number of patients experienced recurrent miscarriages, without successful delivery of a baby [13,14,15] This method could be a viable option in cases with recurrent loses and an exit strategy before performing hysterectomy. Perfusion computed tomography may be a good imaging study for evaluating perfusion characteristics of the transplanted uterus in case of recurrent pregnancy losses. 

Following the successful birth of the baby from our first UTx recipient, we performed the second case from deceased donor and the recipient delivered a healthy baby. In comparison to the first transplant, we were able to achieve pregnancy in a shorter period. As the experience in this field expands, in our opinion, the groups dealing with UTx could expect to achieve pregnancy easier and in a shorter time period. Hence, regarding the UTx, it is very important to publish all data and experiences, either positive or negative.

One of the drawbacks of our case may be the relatively long follow-up of the recipient for nine years without removal of the uterus. Potentially, the risk of nephrotoxicity and malignancy increases as the total duration of immunosuppression increases. However, for young recipients undergoing renal transplant, the risk of a future malignancy is comparable to normal population, especially for women younger than 30 years of age with a total duration of immunosuppression less than 10 years [16,17]. For the keeping of the donor uterus, removal of the womb was suggested to the recipient and her spouse after recurrent pregnancy losses. All possible risks related to keeping the graft for a relatively long time were discussed with all aspects but the patient decided to keep the womb and we respected her autonomous decision. The patient reported strong positive feelings about the experiencing menstrual cycles despite previous pregnancy losses. 

After the performance of the world’s first surgically successful UTx, the studies and trials in this subject accelerated significantly, and the concept of UTx turned into a clinical reality that was once just an experimental surgery in animal models. Particularly following the birth of the baby from this successful transplant, our case was cited by other as the world’s first successful UTx [18].

## 8. Conclusions

UTx using deceased donor uterus is a feasible option for the women suffering from uterus-related infertility. The real success in UTx trials is to achieve live birth with a healthy mother. We believe that, with our contribution to UTx surgery, the studies in this field accelerated and the UTx became a reality rather than an experimental concept. Moreover, one of the most important contributions of our case is the regional vascular augmentation by arterial or venous supercharging, which led to a live birth and remains a viable exit strategy in case of recurrent pregnancy loses in patients with UTx.

## Data Availability

All the data generated or analyzed during this study are included in this published article.
